# Astragalus polysaccharide attenuates metabolic memory-triggered ER stress and apoptosis via regulation of miR-204/SIRT1 axis in retinal pigment epithelial cells

**DOI:** 10.1042/BSR20192121

**Published:** 2020-01-21

**Authors:** Qing-Hua Peng, Ping Tong, Li-Min Gu, Wen-Jie Li

**Affiliations:** 1 Department of Ophthalmology and Otorhinolaryngology in Chinese Medicine, Hunan University of Chinese Medicine and Hunan Key Laboratory, Changsha 410208, P.R. China; 2 Department of Ophthalmology, The Second Xiangya Hospital, Central South University, Changsha 410011, P.R. China; 3 Department of Ophthalmology, Third Affiliated Hospital of Second Military Medical University, Shanghai 200438, P.R. China; 4 College of Integrated Traditional Chinese and Western medicine of Hunan University of Chinese Medicine, Changsha 410013, P.R. China

**Keywords:** Apoptosis, Astragalus polysaccharides, ER stress, Metabolic memory, miR-204, SIRT1

## Abstract

Background:** ‘**Metabolic memory’ of early hyperglycaemic environment has been frequently suggested in the progression of diabetic retinopathy (DR). Retinal pigment epithelial (RPE) cells are crucial targets for DR initiation following hyperglycaemia. Astragalus polysaccharides (APS) has been long used as a traditional Chinese medicine in treating diabetes. In the present study, the preventive effects and mechanisms of APS on metabolic memory-induced RPE cell death were investigated.

Methods: The expressions of miR-204 and SIRT1 were determined by reverse transcription quantitative PCR (RT-qPCR). Dual luciferase assay was applied to detect the potential targeting effects of miR-204 on SIRT1. SIRT1, ER stress and apoptosis related proteins were monitored using Western blotting. Apoptosis was assessed by TUNEL assay and Annexin V/PI staining followed by flow cytometry analysis. MiR-204 mimics and shSIRT1 were applied for miR-204 overexpression and SIRT1 knockdown, respectively.

Results: High glucose exposure induced metabolic memory, which was accompanied with sustained dysregulation of miR-204/SIRT1 axis, high level of ER stress and activation of apoptotic pathway even after replacement with normal glucose. Pre-treatment with APS concentration-dependently reversed miR-204 expression, leading to disinhibition of SIRT1 and alleviation of ER stress-induced apoptosis indicated by decreased levels of p-PERK, p-IRE-1, cleaved-ATF6, Bax, cleaved caspase-12, -9, -3, and increased levels of Bcl-2 and unleaved PARP. The effects of APS on RPE cells were reversed by either miR-204 overexpression or SIRT1 knockdown.

Conclusions: We concluded that APS inhibited ER stress and subsequent apoptosis via regulating miR-204/SIRT1 axis in metabolic memory model of RPE cells.

## Introduction

Diabetic mellitus (DM) exhibits high prevalence worldwide with hyperglycaemia as a hallmark. The phenomenon of ‘metabolic memory’ or ‘hyperglycaemic memory’ has been defined to emphasize the long-lasting diabetic vascular stress by early hyperglycaemic environment [1], and the detrimental effects of metabolic memory persistence even after returning to normal glucose. Early hyperglycaemic exposure is remembered in the target organs including eyes, kidneys, heart and extremities. Among the common diabetic complications, diabetic retinopathy (DR) is a leading cause of global blindness [2]. DR is a progressive disease resulting in loss or dysfunction of photoreceptors or retinal pigment epithelial cells (RPEs) with hyperglycaemia as the main initiator [3–4]. Strong evidence regarding the involvement of metabolic memory during progression of DR was demonstrated by two clinical trials such as Diabetes Control and Complications Trial (DCCT) and Epidemiology of Diabetes Interventions and Complications (EDIC) study [5]. Compared with patients who received intensive therapy, patients who received conventional therapy (dietary control) in DCCT had a higher risk of progressive retinopathy in EDIC, even though blood glucose had been tightly controlled in EDIC [5]. It was therefore suggested that glycemic control plus additional treatment targeting metabolic memory would be beneficial for patients with Type 1 or Type 2 diabetes and its complications [6]. In this regard, novel strategies that may reverse or modulate metabolic memory would hold major potential for clinical therapy in DR.

Metabolic memory is associated with genomic methylation and the increased intracellular advanced glycation end production (AGEs), which leads to activation of NF-κB for up to several months in preclinical models. This in turn causes continuous transcriptome changes, leading to inflammatory reaction, endoplasmic reticulum stress (ER stress), cell apoptosis and consequently the progression of DR [7]. Recently, aberrant retinal miRNA expression profiles have been closely associated with the development of DR. Among 17 miRNAs with altered expression in the retinas of DM rat model, miR-204 was significantly up-regulated [8]. MiR-204 is preferentially expressed in ocular tissues, including fetal human retinal pigment epithelium tissues. Additionally, miR-204 has been revealed to play important roles in the development and function of retinas [9–10]. Previous studies reported that miR-204 might directly target and down-regulate Sirtuin1 lysine deacetylase (SIRT1) in gastric cancer cells and endothelial cells [11–12]. In fact, overexpression of miR-204 was sufficient to induce ER stress accompanying with the reduction of SIRT1 in the endothelial cells. On the other hand, overexpression of SIRT1 could abolish ER stress induced by miR-204 [12]. Nonetheless, whether miR-204 might target SIRT1 and therefore contribute to metabolic memory in PRE cells has not been reported. It is postulated that up-regulation of miR-204 in the DR model might reduce SIRT1 expression and cause metabolic memory that is related with ER stress as well as subsequent apoptosis.

Astragalus polysaccharides (APS) is a bioactive polysaccharide extracted from the root of Astragalus membranaceus (Huang qi), a traditional Chinese Medicine, which has been widely applied in clinic for its anti-tumor activities and anti-diabetic properties [13–14]. Previous *in vivo* studies found that APS treatment could lower the incident rate and postpone the onset of Type 1 and Type 2 diabetes [14]. It was also reported that APS could inhibit ER stress and subsequent apoptosis. Importantly, not only had glucose homeostasis been restored, but the important leading factor ER stress had also been reduced in the liver of rat model of Type 2 diabetes after APS treatment [15]. These suggested that APS had a functional role in glycaemic regulation and insulin-resistance inhibition. However, the effects of APS on metabolic memory in retinal pigment epithelial cells have not been reported.

In this article, we investigated the prevention mechanisms of APS in metabolic memory-triggered ER stress and subsequent apoptosis in retinal pigment epithelial cells. We found that APS functioned to up-regulate SIRT1 in high glucose-induced diabetic retinopathy and metabolic memory models via inhibiting miR-204 and subsequent ER stress as well as apoptosis. For the first time, we highlighted the pathogenesis of metabolic memory about miR-204/SIRT1 axis and the potential of APS in drug development on metabolic memory-mediated diabetic retinopathy.

## Materials and methods

### Regents and antibodies

APS was purchased from Medchem express (Monmouth Junction, NJ, U.S.A.). APS was dissolved in DMSO and diluted to working solution with culture medium in 5 mM glucose before use. Primary antibodies against SIRT1 (#8469), Protein kinase R-like endoplasmic reticulum kinase (PERK, #5683), p-PERK (Thr980, #3179), Inositol-requiring enzyme 1 (IRE1, #3294), cleaved activating transcription factor 6 (ATF6, #65880), caspase-3 (#9664), -9 (#52873), -12 (#2202), PARP (#9542), Bcl-2 (#15071), Bax (#5023) and GAPDH (#5174) and secondary antibodies (HRP linked anti-mouse, #7076; HRP linked anti-rabbit, #7074; Alexa Fluor® 488 conjugated anti-rabbit, #4412) were purchased from Cell signaling technology (Danvers, MA, U.S.A.). Anti-phosphorylated IRE-1 (Ser724, #PA-16927) was the product of Thermo Fisher Scientific (San Jose, CA, U.S.A.). The transfection reagent, Lipofectamin 2000, was purchased from Invitrogen. The Annexin V-FITC apoptosis detection kit was obtained from Becton-Dickinson (Franklin Lakes, NJ, U.S.A.). TUNEL apoptosis detection kit was ordered from KeyGEN BioTECH (Jiangsu, CN). ProLong Diamond Antifade mounting reagent with DAPI, protease inhibitor tablets and Pierce BCA protein assay kit were purchased from ThermoFisher Scientific (San Jose, CA, U.S.A.). PrimeScript RT reagent Kit and SYBR Premix Ex Taq II were ordered from Takara (Dalian, CN).

### Isolation primary rat RPE cells

The animal study was approved by the Guidelines for the Care and Use of Laboratory Animals of in Human University of Chinese Medicine. Isolation of rat primary retinal pigment epithelial (PRPE) cells was performed as previously described [16]. Briefly, healthy male rats were used for PRPE cells harvest and culture. Extraocular tissues were removed from freshly enucleated eyes. A cut originated from the optic nerve was made and then three additional radial incisions were made with a scalpel. The eye was then incubated in a 24-well plate containing 20 U/ml papain solution (Worthington PDS Kit, Lakewood, NJ, U.S.A.) for 1 h at 37°C. The eyes were then transferred to DMEM supplemented with 10% FBS. An incision along the ora serrata was made to remove the lens and cornea-iris. The retina/RPE complex was then pulled out and digested in 1 ml of 20 U/ml papain solution for 10 min at 37°C. The PRPE cells were separated from the retina, incubated and triturated in 1 mg/mL trypsin (Sigma-Aldrich, St.Louis, MO, U.S.A.). The trypsinized cells were washed and centrifuged in DMEM supplemented with 10% FBS. The PRPE cells were then ready for seeding.

### Cell culture

The human RPE cell line (ARPE-19, Shanghai GuanDao Biotech Co., Ltd., Shanghai, China) was cultured in Dulbecco’s modified Eagle’s medium and F-12 nutrient mixture (Hyclone, Logan, UT, U.S.A.), supplemented with 10% FBS (Gibco, Grand Island, NY, U.S.A.) and penicillin (100 U/ml)/streptomycin (100 μg/ml) (Sigma-Aldrich, St.Louis, MO, U.S.A.). Cells were cultivated at 37°C in a humidified atmosphere of 5% CO_2_. Cells that had grown to 80% confluence were used in the experiments. For primary RPE cells, the PRPE cells isolated from retina were seeded into matrigel (BD Biosciences, San Jose, CA, U.S.A.) treated plates and cultured by ‘Miller medium’ (DMEM, N1 medium supplement, MEM-non-essential amino acids, 2 mM GlutaMAX™-I, 250 μg/ml taurine, 20 ng/ml hydrocortisone, 13 ng/ml triiodothyronin and antibiotics) supplemented with 20% FBS. On the next day, medium was changed to ‘Miller medium’ supplemented with 5% FBS. After plating, cells were treated with normal glucose (5 mM, NG group), high glucose (30 mM, HG group), mannitol (30 mM, isotonic control) or temporary high glucose treatment. For ARPE-19 cells, cells in temporary high glucose group were treated with high glucose (30 mM) for 3 days followed by normal glucose (5 mM) for 3 days. For PRPE cells, cells in temporary high glucose group were treated with high glucose for 2 days followed by normal glucose for 4 days. For APS treatment, APS (12.5, 25 and 50 µg/ml) was dissolved in normal glucose condition and added to cells whenever high glucose was replaced with normal glucose (Supplementary Figure S1).

### Transfection

MicroRNA oligos and shRNA-expressing pGPH1 plasmids were ordered from GenePharma (Shanghai, CN). For microRNA studies, ARPE-19 cells were seeded onto six-well plate and transfected with 25 pmol of miR-204 mimics, miR-204 inhibitor or their scramble control (NC) using 7.5 µl of Lipofectamine RNAiMAX reagent (Thermo Scientific, San Jose, CA, U.S.A.). For RNA interference studies, pGPH1-shSIRT1 or pGPH1-scramble plasmids (1 µg/well) and Lipofectamine 2000 (3 µl/well) were mixed and applied to ARPE-19 cells in six-well plate.

### RNA extraction and reverse transcription-quantitative polymerase chain reaction (RT-qPCR)

Total RNA was extracted using Trizol reagent (ThermoFisher Scientific, San Jose, CA, U.S.A.) according to manufacturer’s instruction. Complementary DNA was synthesized using the PrimeScript RT reagent Kit. Quantitative-PCR (qPCR) analysis was performed using SYBR Premix Ex Taq II in an ABI 7500 Real-Time PCR system (Applied Biosystems, Foster City, CA, U.S.A.). Mature miR-204 expression was quantified using the TaqMan MicroRNA Reverse Transcription Kit (Applied Biosystems) and TaqMan MicroRNA Assay Kit (Applied Biosystems), with U6 snRNA used as the endogenous control. The relative expression of miR-204 and *Sirt1* was calculated by the 2^−ΔΔ*C*^_t_ method. All the primer sequences for qPCR are as below.

Human miR-204 forward 5′-TTCCCTTTGTCATCCTATGCCT-3′ and reverse 5′- CCAGTCTCAGGGTCCGAGGTATTC-3′;

Human SIRT1 forward 5′-TAGCCTTGTCAGATAAGGAAGGA-3′ and reverse 5′- ACAGCTTCACAGTCAACTTTGT-3′;

Human GAPDH forward 5′-AGAAGGCTGGGGCTCATTTG -3′ and reverse 5′-AGGGGCCATCCACAGTCTTC-3′;

Human U6snRNA forward 5′-AAAGCAAATCATCGGACGACC-3′ and reverse 5′- GTACAACACATTGTTTCCTCGGA-3′;

### Dual luciferase assay

The 3′UTR sequence of *Sirt1* containing predicted miR-204 target sites was amplified by PCR and cloned to the downstream of the firefly luciferase gene in a pmirGLO vector (Promega, Madison, WI, U.S.A.). Site-directed mutagenesis was performed to obtain the mutant *Sirt1* 3′UTR. pRL *Renilla* luciferase plasmid (Promega, Madison, WI, U.S.A.) was applied as the transfection control. Cells were seeded in 48-well plates. For transfection, 100 ng of pmirGLO (wild-type or mutant), 5 ng of pRL and 10 pmol of microRNA oligos (miR-204 mimics or NC) were co-transfected into cells using 0.3 µl of Lipofectamine 2000 reagent according to the instruction of manufacturer. Firefly and *Renilla* luminance were measured using the Dual Luciferase Reporter Assay Kit 48 h after transfection according to the provided protocol (Promega, Madison, WI, U.S.A.).

### Apoptosis assay

For Annexin V/Propidium iodide (PI) assay, ARPE-19 or PRPE cells were plated into 12-well plates at a density of 1 × 10^5^ cells/well. After treatment as described above, cells were dissociated from culture plates and harvested for staining using Annexin V-FITC/PI, according to manufacturer’s instructions. Cells were analyzed by flow cytometry (Becton-Dickinson, Franklin Lakes, NJ, U.S.A.). FITC^+^/PI^−^ fraction and FITC^+^/PI^+^ fraction were considered as apoptotic cells (early and late apoptosis, respectively). For TUNEL assay, the cells were permeabilized with 0.2% Triton X-100 in PBS for 30 min, following by proteinase K digestion for 15 min. After washing with PBS, the cells were labeled with TdT reaction mixture for 1 h at 37°C and visualized under the fluorescence microscope. Apoptotic cells in each group were counted by researchers.

### Western blotting

Total protein was extracted with cell lysis buffer (50 mM Tris, 150 mM NaCl, 1% NP-40, 1 mM EDTA, pH 7.6) containing a cocktail of protease inhibitors. Protein concentration was determined using Pierce BCA protein assay kit (San Jose, CA, U.S.A.) according to manufacturer’s instruction. Thirty microgram protein samples were separated on SDS-polyacrylamide gels, then transferred onto PVDF membranes (0.22 μm pore, Roche). After blocking with TBST buffer (20 mM Tris, 137 mM NaCl, 0.1% Tween-20, pH 8.0) containing 5% non-fat milk, membranes were incubated with primary antibodies (1:1000) overnight at 4°C. Then, membranes were incubated with secondary antibody (1:3000) for 1 h at room temperature. The protein bands were visualized using Immobilon Western Chemiluminescent HRP substrate (Millipore, Burlington, MA, U.S.A.).

### Statistical analysis

All experiments were performed at least three times, with one representative experiment shown. Data were presented as the mean ± standard deviation (S.D.). All statistical analysis was carried out using the SPSS statistical software package (Chicago, IL, U.S.A.). Statistical evaluation was performed using Student’s *t* test (two-tailed) between two groups or one-way analysis of variance (ANOVA) followed by Tukey post hoc test for multiple comparison. *P* < 0.05 was considered statistically significant in all cases.

## Results

### APS reversed high glucose-triggered miR-204 up-regulation and SIRT1 down-regulation in RPE cells

**Figure 1:**
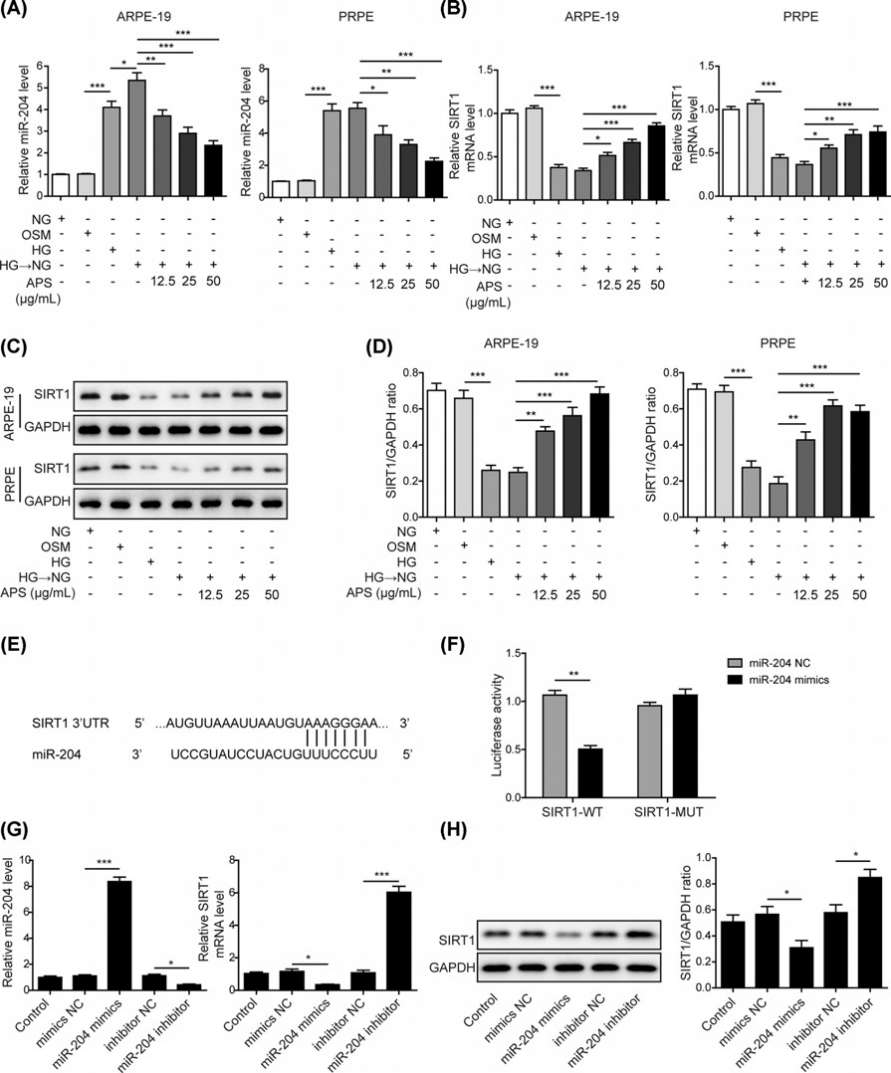
APS reversed high glucose-triggered miR-204 up-regulation and SIRT1 down-regulation in RPE cells (**A**) APS reversed high glucose-induced alteration of miR-204 in ARPE-19 and PRPE cells. (**B**) APS reversed high glucose-induced alteration of SIRT1 in ARPE-19 and PRPE cells. (**C**) Metabolic memory-induced reduction of SIRT1 protein was reversed by APS in a concentration-dependent manner. (**D**) Quantification results in panel (C). (**E**) SIRT1 3′UTR contains a putative binding site for miR-204 seed sequence. (**F**) Dual luciferase assay confirmed SIRT1 was a direct target of miR-204. (**G**) MiR-204 mimics transfection led to increase of miR-204, whereas miR-204 inhibitor efficiently reduced miR-204 expression. This was accompanied with SIRT1 down-regulation by mimics and SIRT1 elevation by inhibitor. (**H**) Western blotting assay confirmed that miR-204 mimics caused SIRT1 reduction. By contrast, miR-204 inhibitor transfection led to SIRT1 increase; HG, high glucose (30 mM); NG, normal glucose (5 mM); OSM, isotonic control group. HG-NG, high glucose (30 mM) followed by normal glucose (5 mM). The result was a representative of three independent experiments. Error bars represented mean ± SD. *P* values were determined by one-way analysis of variance (ANOVA) followed by Tukey post hoc test; *P < 0.05, **P < 0.01 and ***P < 0.001.

We performed RT-qPCR and Western blotting to examine the functions of APS on regulating miR-204 and its target SIRT1 in human retinal pigment epithelial cells (ARPE-19) and rat primary retinal pigment epithelial (PRPE) cells. High glucose (30 mM) incubation for 6 days significantly increased the expression of miR-204 but reduced the mRNA and protein levels of SIRT1 when compared with normal glucose (NG) and mannitol exposure (Figure 1A–D). Notably, the up-regulation of miR-204 and down-regulation of SIRT1 sustained, even after replacement with NG on the day 2 or day 3 (Figure 1A–D). This finding suggested metabolic memory of high glucose was induced. However, pre-treatment with APS caused a concentration-dependent inhibition of miR-204 expression while restored the mRNA and protein levels of SIRT1 in HG-NG treated-ARPE-19 cells and PRPE cells (Figure 1A–D). As miR-204 was predicted to have a conserved seed match at 384-391 position on *Sirt1* 3′UTR (Figure 1E), we examined the impact of miR-204 on *Sirt1* 3′UTR activity by dual luciferase assay. Luciferase activity was inhibited by miR-204 mimics in cells expressing wild-type *Sirt1* 3′-UTR but not in cells transfected with mutant counterpart (Figure 1F). Furthermore, the mRNA and protein levels of SIRT1 were up-regulated or down-regulated in the presence of miR-204 inhibitor or mimics, respectively (Figure 1G,H). This confirmed that SIRT1 was a direct target of miR-204. Taken together, cellular metabolic memory of high glucose altered the miR-204/SIRT1 axis, which could be reversed by APS administration.

### APS inhibited high glucose-induced ER stress in RPE cells

**Figure 2:**
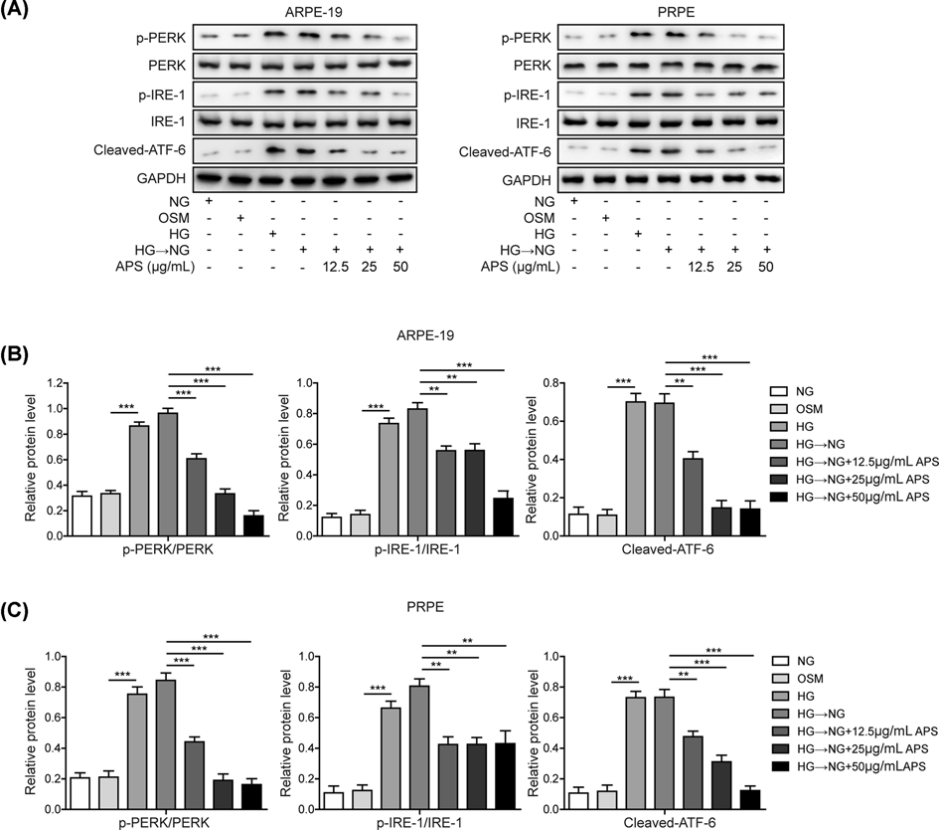
APS inhibited high glucose-induced ER stress in RPE cells (**A**) HG exposure caused ER stress in ARPE-19 and PRPE cells. Metabolic memory maintained high level of ER stress (HG-NG). Pre-treatment with APS suppressed metabolic memory-triggered ER stress in a concentration-dependent manner. (**B** and** C**) Quantification results in panel (A); HG, high glucose (30 mM); NG, normal glucose (5 mM); OSM, isotonic control group. HG-NG, high glucose (30 mM) followed by normal glucose (5 mM). The result was a representative of three independent experiments. Error bars represented mean ± SD. *P* values were determined by one-way analysis of variance (ANOVA) followed by Tukey post hoc test; **P < 0.01 and ***P < 0.001.

ER stress plays key roles in the complications of DR. As SIRT1 was a critical modulator of ER stress, we examined whether high glucose-induced ER stress could be affected by APS treatment. To access the ER stress, the levels of p-PERK, PERK, p-IRE-1, IRE-1 and cleaved ATF6 were detected by Western blotting [17–18]. HG treatment triggered ER stress as indicated by the increase of p-PERK, p-IRE-1 and the cleaved form of ATF-6 (Figure 2A–C). No significant difference between HG and HG-NG was observed, indicating that ER stress was preserved upon metabolic memory induction by initial HG exposure. However, pre-treatment with APS efficiently prevented high glucose-induced ER stress and reversed metabolic memory-related ER stress in a concentration-dependent manner (Figure 2A–C).

### APS inhibited high glucose-induced apoptosis in RPE cells

**Figure 3:**
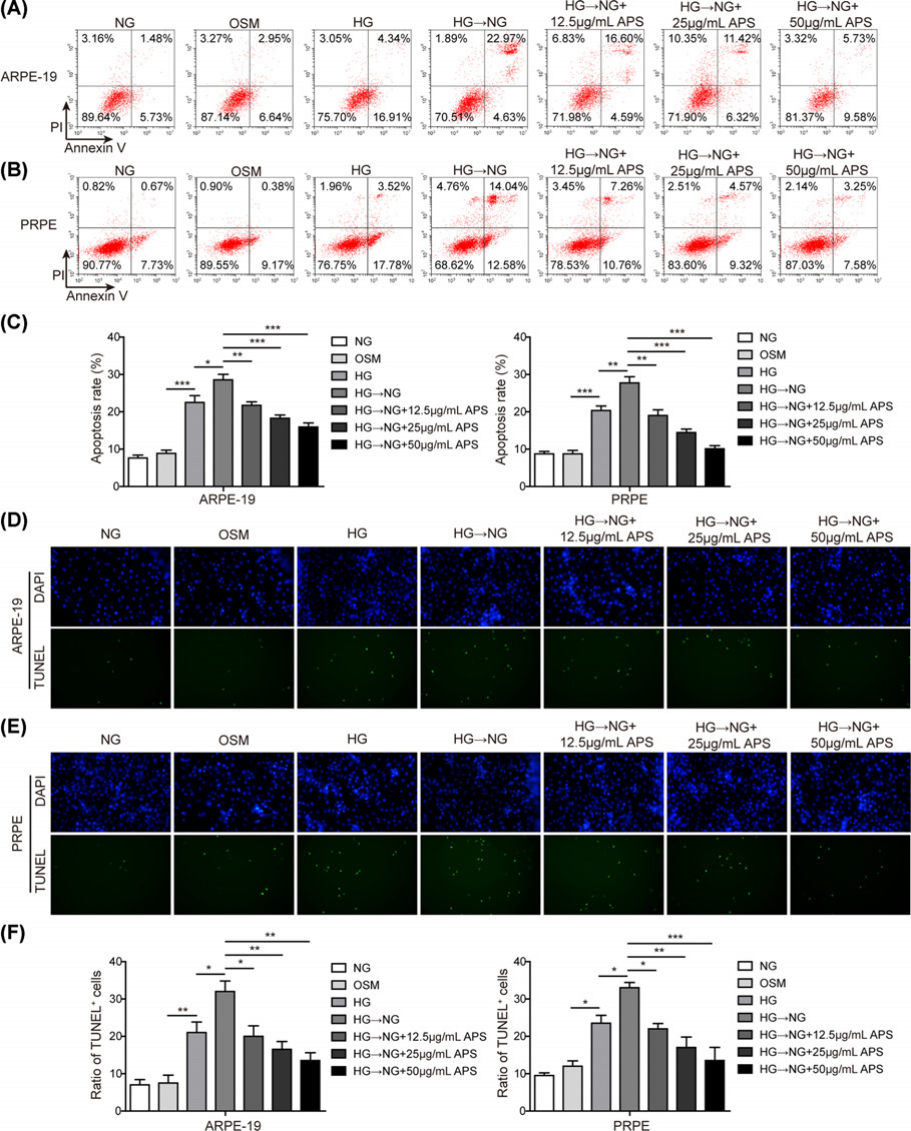
APS inhibited high glucose-induced apoptosis in RPE cells (**A** and** B**) Apoptosis was determined by Annexin V-FITC/PI staining with flow cytometry in ARPE-19 (A) and PRPE (B) cells. FITC positive cells (right upper and right lower quadrants) were considered as apoptotic cells. (**C**) Statistical results in panel (A). (**D** and **E**) Apoptosis was detected using TUNEL assay in ARPE-19 (D) and PRPE (E) cells. Cells labeled with TUNEL (green signal) were considered as apoptotic cells. Apoptotic cells were counted and compared among groups. (**F**) Statistical results in panels (D and E). HG, high glucose (30 mM). NG, normal glucose (5 mM). OSM, isotonic control group. HG-NG, high glucose (30 mM) followed by normal glucose (5 mM). The result was a representative of three independent experiments. Error bars represented mean ± SD. *P* values were determined by one-way analysis of variance (ANOVA) followed by Tukey post hoc test; *P < 0.05, **P < 0.01 and ***P < 0.001.

**Figure 4:**
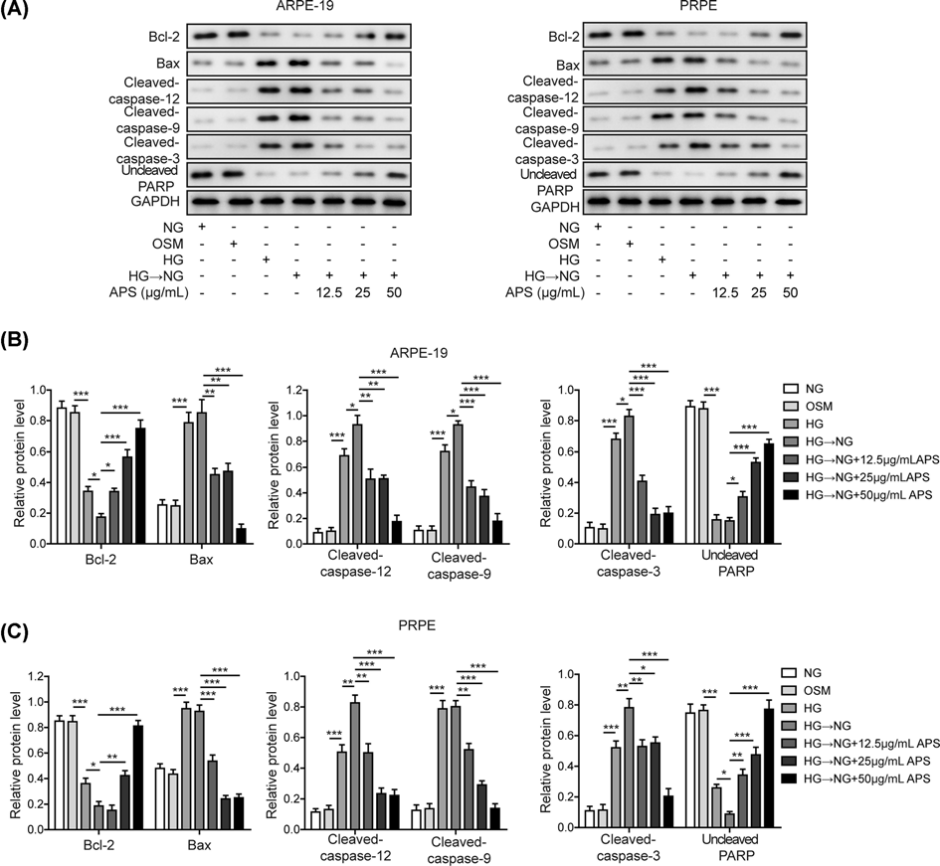
APS effects on apoptosis-related proteins in RPE cells (**A**) Apoptotic markers were evaluated in ARPE-19 and PRPE cells. The anti-apoptosis protein Bcl-2 and uncleaved PARP were down-regulated, while pro-apoptotic markers Bax, cleaved form of caspase-12, caspase-9 and caspase-3 were up-regulated after treatment with HG or HG-NG. Pre-treatment with APS reversed these changes in a concentration-dependent manner. (**B** and **C**) Quantification results in panel (A). HG, high glucose (30 mM). NG, normal glucose (5 mM). OSM, isotonic control group. HG-NG, high glucose (30 mM) followed by normal glucose (5 mM). The result was a representative of three independent experiments. Error bars represented mean ± SD. *P* values were determined by one-way analysis of variance (ANOVA) followed by Tukey post hoc test; *P < 0.05, **P < 0.01 and ***P < 0.001.

Increased ER stress in the retina is frequently associated with apoptosis induction [19]. We then investigated whether APS might prevent RPE cells from apoptosis. Annexin V/PI staining was performed to determine the apoptotic rate. As expected, HG-NG treatment significantly increased the number of apoptotic cells, when compared with mannitol-treated group (about 21.52% vs 9.59% in APRE-19 and 21.3% vs 9.55% in PRPE). However, HG-NG treatment caused comparable apoptosis ratio as HG treatment. Treatment with APS significantly reduced the rate of apoptosis at all concentrations tested (Figure 3A–C). Notably, high concentration of APS (50 µg/ml) exhibited strong protective effects by reducing to about 15% or 11% of its apoptosis ratio. Similar results were observed in TUNEL staining assay. The ratio of TUNEL positive cells from each group was counted. As shown in Figure 3D–F, both HG and HG-NG significantly increased TUNEL^+^ cells of APRE-19 and PRPE. Pre-treatment with different concentrations of APS prevented the increase of the ratio of TUNEL positive cells in a concentration-dependent manner. Further, apoptotic markers were also assessed in both cells by Western blotting. Both HG and HG-NG treatment caused a series of changes in apoptosis-related markers, i.e. decreased Bcl-2 level, increased Bax level and enhanced cleavage of capase-9, -3 as well as PARP (Figure 4A–C). Moreover, it should be noted that cleaved casepase-12 was also significantly increased, indicating that ER stress was attributed to the apoptosis induction. APS pre-treatment, however, prevented such changes in a concentration-dependent manner. Our data showed that APS reversed metabolic memory-related apoptosis via caspase-12 dependent pathway in RPE cells.

### MiR-204 mimics and shSIRT1 abolished the APS effects on miR-204 and SIRT1 expression levels in APRE-19 cells

**Figure 5:**
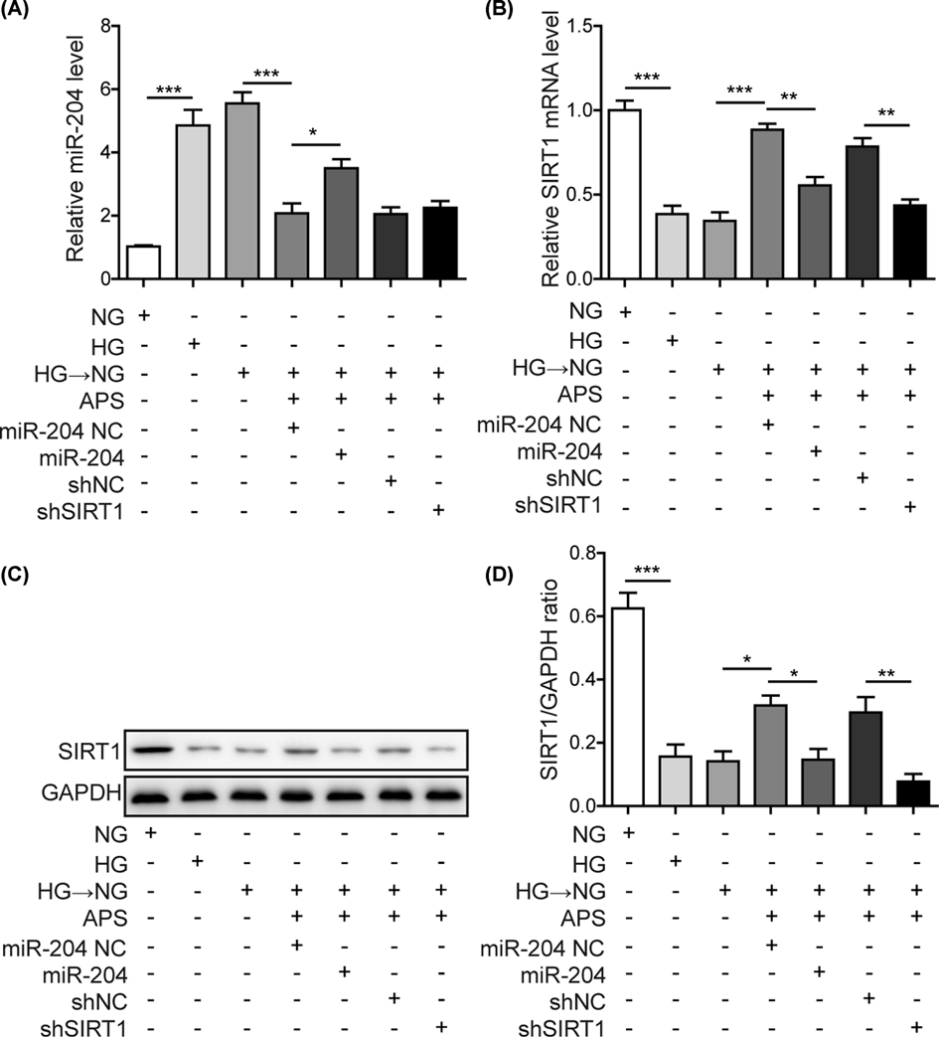
MiR-204 mimics and shSIRT1 abolished the APS effects on miR-204 and SIRT1 expression levels in APRE-19 cells (**A**) HG and HG-NG exposure significantly up-regulated miR-204, while APS reversed this effect as above. Transfection with miR-204 mimics refilled intracellular miR-204 level. Knockdown of SIRT1 had no effects on miR-204 expression. (**B**) APS recovered SIRT1 level by HG-NG treatment. This effect was abolished by the miR-204 mimics and shSIRT1 transfection. (**C**) The alteration of SIRT1 protein was consistent with mRNA detection. (**D**) Quantification results of panel (C). HG, high glucose (30 mM). NG, normal glucose (5 mM). OSM, isotonic control group. HG-NG, high glucose (30 mM) followed by normal glucose (5 mM). The result was a representative of three independent experiments. Error bars represented mean ± SD. *P* values were determined by one-way analysis of variance (ANOVA) followed by Tukey post hoc test; *P < 0.05, **P < 0.01 and ***P < 0.001.

We next investigated the mechanisms of APS in regulating ER stress and apoptosis. The ARPE-19 cells were transfected with miR-204 mimics and shSIRT1, respectively. As shown in Figure 5A, miR-204 mimics significantly replenished the loss of miR-204 expression in HG-NG group induced by APS. Meanwhile, the recovery of SIRT1 expression by APS was completely abolished (Figure 5B). Transfection with shSIRT1 had no effect on miR-204 expression, and SIRT1 mRNA level induced by APS was significantly decreased by shSIRT1 (Figure 5B). Western blotting confirmed the alteration pattern observed in SIRT1 mRNA (Figure 5C and D). These results indicated that miR-204 mimics reversed the APS-induced down-regulation of miR-204 and the up-regulation of its direct downstream target SIRT1.

### MiR-204 mimics and shSIRT1 reversed the APS effects on ER stress and apoptosis in APRE-19 cells

**Figure 6:**
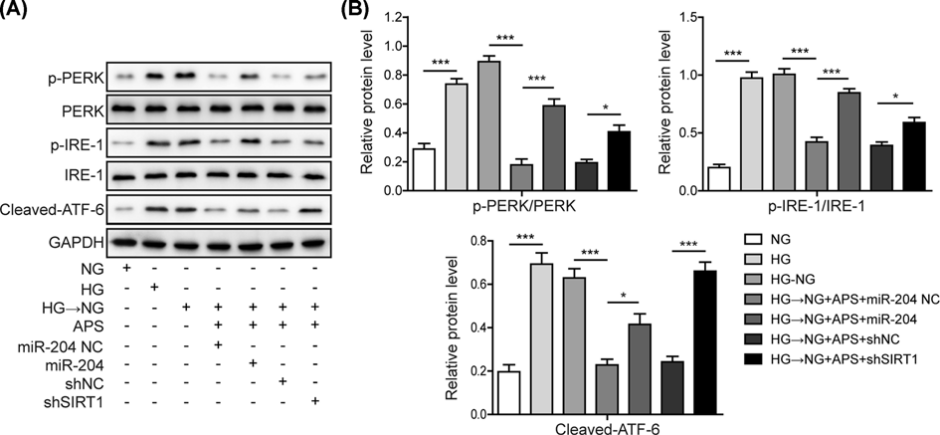
MiR-204 mimics and shSIRT1 reversed the APS effects on ER stress in APRE-19 cells (**A**) ER stress was evaluated by determining the phosphorylated PERK, phosphorylated IRE-1 and the cleaved ATF-6. (**B**) Quantification results of panel (A). HG, high glucose (30 mM). NG, normal glucose (5 mM). OSM, isotonic control group. HG-NG, high glucose (30 mM) followed by normal glucose (5 mM). The result was a representative of three independent experiments. Error bars represented mean ± SD. *P* values were determined by one-way analysis of variance (ANOVA) followed by Tukey post hoc test; *P < 0.05 and ***P < 0.001.

**Figure 7:**
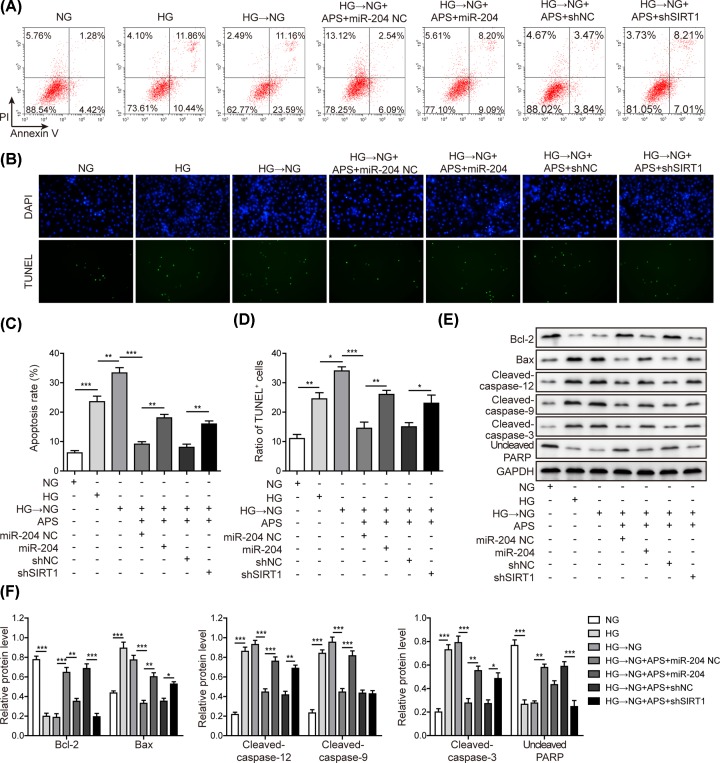
MiR-204 mimics and shSIRT1 reversed the APS effects on apoptosis in APRE-19 cells (**A**) HG-NG caused a significant elevation of apoptosis as indicated by flow cytometry (FITC positive cells in right upper and lower quadrants). APS pre-treatment efficiently prevented metabolic memory-induced ARPE-19 cells apoptosis. Overexpression of miR-204 or knockdown of SIRT1 abolished the preventive actions of APS. (**B**) TUNEL assay was performed in ARPE-19. (**C**) Quantification results of panel (A). (**D**) Quantification results of panel (B). (**E**) Apoptotic markers were detected and compared after different treatment. APS restored metabolic memory-induced Bcl-2 reduction. However, miR-204 overexpression or SIRT1 knockdown abolished the action of APS. Pro-apoptotic markers were suppressed by APS pre-treatment, which was also abolished by miR-204 mimics or shSIRT1 transfection. (**F**) Quantification results of panel (E). HG, high glucose (30 mM). NG, normal glucose (5 mM). OSM, isotonic control group. HG-NG, high glucose (30 mM) followed by normal glucose (5 mM). The result was a representative of three independent experiments. Error bars represented mean ± SD. *P* values were determined by one-way analysis of variance (ANOVA) followed by Tukey post hoc test; *P < 0.05, **P < 0.01 and ***P < 0.001.

To investigate the role of miR-204/SIRT1 axis in APS-mediated protective effects, miR-204 overexpression or SIRT1 knockdown was performed. It was shown that miR-204 mimics or shSIRT1 reversed the decreased levels of p-PERK, p-IRE-1 and clevaved-ATF-6 in ARPE-19 cells that were inhibited by APS (Figure 6A,B). This suggested overexpression of miR-204 or knockdown of SIRT1 was sufficient to reverse the effects of APS on high glucose-induced ER stress. Apoptotic rates were also examined. Percentage of apoptotic cells suppressed by APS was increased again by miR-204 mimics or shSIRT1 treatment, as indicated by Annexin V/PI staining (Figure 7A,C) and TUNEL assay (Figure 7B,D). Moreover, the effects of APS on Bcl-2, Bax, caspase-12, -9, -3 and PARP were completely abolished by transfection with miR-204 mimics or shSIRT1 (Figure 7E,F). Taken together, the data demonstrated that APS prevented RPE cells from ER stress and apoptosis and regulated metabolic memory via modulating miR-204/SIRT1 axis.

## Discussion

DR is intimately linked to diabetes mellitus and remains the leading cause of preventable vision loss in adults aged 20–74 year [20]. In the USA, it is estimated that 40% of Type 1 diabetic patients and up to 86% of Type 2 diabetic patients have DR [21]. While research continues to broaden the pathological understanding of DR, investigations in advanced medicines are expected to emerge. As studies have suggested that metabolic memory contributes to the pathogenesis of DR, so regulating metabolic memory is one of the feasible therapeutic options against DR progression. Glycaemic memory-induced ER stress and apoptosis play critical roles in the progression of DR. Therefore, the development of relevant medicines that have anti-ER stress and anti-apoptosis functions has potential to offset the deleterious effects of high glucose exposure, thus retarding the progression of DR. APS are the major phytoconstituents in *Astragalus membranaceus* that has been addressed to exhibit anti-diabetic properties [14]. However, the exact effects on preventing or treating metabolic memory and DR and underlying mechanisms of APS are unknown. In the current study, we reported that APS treatment reversed miR-204 elevation upon metabolic memory, leading to disinhibition of SIRT1 expression and prevention of ER stress as well as apoptosis in RPE cells.

RPE cells are essential for neuroretina survival and visual function. Also, structural and functional abnormalities of RPE cells have been found in DR [22]. Therefore, we used high glucose-stimulated RPE cells to represent cellular model of DR. We proposed that APS treatment regulated miR-204/SIRT1 axis in progression of metabolic memory-induced DR. We demonstrated hyperglycaemia exposure led to the up-regulation of miR-204 and down-regulation of SIRT1 in ARPE-19 and PRPE cells. This was sustained after replacement with NG on the day 2 or 3. However, pre-treatment of APS reversed this regulation on miR-204 and SIRT1 in a concentration-dependent manner. Taken together with the observation that miR-204 directly binded to SIRT1 3′UTR to negatively regulate expression of SIRT1, our data suggested that APS exerted potent regulatory effect on miR-204/SIRT1 axis in RPE cells. In previous study, APS attenuated insulin resistance through regulating miR-203a-3p in Type 2 diabetic mellitus [23]. In addition, APS treatment regulated SIRT1 pathway to improve insulin resistance [24] and ameliorate mitochondria dysfunction [25]. The present study reflected that APS treatment also regulated SIRT1 pathway in high glucose-induced cellular model of metabolic memory via inhibition of miR-204. We are the first group to observe the effects of APS on the miR-204/SIRT1 axis in metabolic memory and DR.

Down-regulation of SIRT1 critically promoted ER stress [12]. Furthermore, recent studies suggested that ER stress was one of the causative factors in the pathogenesis of DR. Mammalian cells activate unfolded protein response (UPR) to recover or maintain normal ER function [26]. While in long term, UPR activation switches into a pro-apoptotic role [27]. Activation of UPR results in accumulating ER stress in diabetic retina to promote major pathophysiological events including apoptosis [28]. In the present study, we observed high glucose treatment induced ER stress and subsequent apoptosis in APRE-19 and PRPE cells. This was sustained when high glucose was replaced with normal glucose, indicating that the metabolic memory contributed to the ER stress and subsequent apoptosis induction. We showed that the induction of p-PERK, P-IRE1 and cleaved ATF-6 were inhibited by APS in a concentration-dependent manner at the protein level. Further, we found that the increase in number of apoptotic cells, as well as the repression of Bcl-2, uncleaved PARP, and the induction of Bax, cleaved caspase-12, -9, -3 induced by high glucose, were reversed with APS treatment in a concentration-dependent manner. These indicated that treatment of APS inhibited metabolic memory-resulted ER stress and subsequent apoptosis. The apoptosis was at least partly resulted from ER stress because of the enhancement of caspase-12. Previously, APS was demonstrated to modulate a series of signaling pathways in cells insulted with high glucose, including AMPK, SIRT-PGC1α/PPARα-FGF21, PPARγ, PI3K/AKT, oxidative stress, intrinsic apoptosis, extrinsic apoptosis and mitochondria injury [24,29–32]. Our results mainly suggested a novel mechanism that APS was able to mediate its effect on ER stress and apoptosis of RPE cells with glycaemic memory, highlighting its potential in preventing DR progression. Also, the above other signaling pathways that were not investigated in the present study due to the time limitation are worthy being deeply explored and performed in our lab in the future study. These signaling pathways will provide some other potential mechanisms and therapeutic strategies about metabolic memory and DR.

Based on these observations, APS was likely to attenuate ER stress and subsequent apoptosis in RPE cells with hyperglycaemic memory. A possible mechanism for this function could be the regulatory effect of APS on miR-204/SIRT1 axis. To provide more sophisticated mechanistic details of the effects of APS on RPE cells in hyperglycaemic condition, we transfected miR-204 mimics for miR-204 overexpression and shSIRT1 for SIRT1 knockdown. We found that miR-204 mimics and shSIRT1 transfection were able to reverse the effects of APS on down-regulation of miR-204 and subsequent up-regulation of SIRT1 expression. This further added clues that APS exerted its effect in RPE cells via miR-204/SIRT1 axis. Transfection of miR-204 mimics or shSIRT1 into hyperglycaemia-treated RPE cells reversed the attenuation of ER stress and subsequent apoptosis following APS treatment. As p-PERK, p-IRE-1 and cleaved ATF-6 expression levels were disinhibited in the miR-204 mimics or shSIRT1 transfected APS-treatment group, it implied that miR-204/SIRT axis played a role in regulation of APS on ER stress and the subsequent apoptosis in RPE cells.

While APS mediating miR-204 down-regulation was one of the key findings, the lack of underlying molecular mechanism was the limitation of the current study. Previous studies revealed that abnormal miR-204 expression was correlated to several pathological conditions of human diseases [33,34] and rodent disease models [12,35], indicative of dysregulated transcriptional modulation of miR-204 in diseases. Meanwhile, it was demonstrated that long non-coding RNA or circular RNA may act as competing endogenous RNAs (ceRNAs) to alter the expression level of miR-204 [36–38]. ceRNA sponges therefore represent an alternative mechanism of miR-204 regulation. Accumulating reports have highlighted the roles of APS in regulating miRNA expression, for example, APS up-regulated or maintained the miR-203a-3p expression levels to attenuate insulin resistance of Type 2 diabetes mellitus [23]. APS suppressed miR-721 expression to attenuate TNF-α-induced insulin resistance in 3T3-L1 adipocytes [30]. Dietary APS supplements could regulate testicular miRNA expression profiles [39]. But, none of these studies have explicitly demonstrated whether APS modulated miRNA expression by directly participating in the transcription, or indirectly by regulating miRNA sponges. It is not clear and definite how APS regulates the expression of miRNA in the currently published researches. It is intriguing to investigate the mechanism of APS-mediated miRNA expression, including miR-204, which is the main direction of our follow-up studies. The other limitation of the current report is that no animal studies have been involved. However, the findings of miR-204/SIRT1 axis and ER stress on metabolic memory will shed light on the directions of animal models in the future. We fully agreed that *in vivo* studies would definitely strengthen the conclusion drew by current data.

## Supplementary Material

Supplementary Figure S1

## Abbreviations

APS: astragalus polysaccharides
DM: diabetic mellitus
DR: diabetic retinopathy
RPE: retinal pigment epithelial
RT-qPCR: reverse transcription quantitative PCR
SIRT1: Sirtuin1 lysine deacetylase

## References

[1] Ceriello A. (2012) The emerging challenge in diabetes: The “metabolic memory”. Vascul. Pharmacol. 57, 133–138 10.1016/j.vph.2012.05.005 22609133

[2] Sabanayagam C., Yip W., Ting D.S.W., Tan G. and Wong T.Y. (2016) Ten Emerging Trends in the Epidemiology of Diabetic Retinopathy. Ophthalmic Epidemiol. 23, 209–222 10.1080/09286586.2016.1193618 27355693

[3] Gubitosi-Klug R.A. (2014) The Diabetes Control and Complications Trial/Epidemiology of Diabetes Interventions and Complications Study at 30 Years: Summary and Future Directions: Figure 1. Diabetes Care 37, 44–49 10.2337/dc13-2148 24356597 PMC3867991

[4] Frank R.N. (2004) Diabetic retinopathy. N. Engl. J. Med. 350, 48–58 10.1056/NEJMra021678 14702427

[5] Writing Team for the Diabetes Control and Complications Trial/Epidemiology of Diabetes Interventions and Complications Research Group (2003) Sustained Effect of Intensive Treatment of Type 1 Diabetes Mellitus on Development and Progression of Diabetic Nephropathy. JAMA 290, 2159 10.1001/jama.290.16.2159 14570951 PMC2622725

[6] Ceriello A., Ihnat M.A. and Thorpe J.E. (2009) Clinical review 2: The &quot;metabolic memory&quot;: is more than just tight glucose control necessary to prevent diabetic complications? J. Clin. Endocrinol. Metab. 94, 410–415 19066300 10.1210/jc.2008-1824

[7] Zhang L., Chen B. and Tang L. (2012) Metabolic memory: Mechanisms and implications for diabetic retinopathy. Diabetes Res. Clin. Pract. 96, 286–293 10.1016/j.diabres.2011.12.006 22209677

[8] Wang F.E. et al. (2010) MicroRNA-204/211 alters epithelial physiology. FASEB J. 24, 1552–1571 10.1096/fj.08-125856 20056717 PMC3231816

[9] Wu J. et al. (2012) Altered MicroRNA Expression Profiles in Retinas with Diabetic Retinopathy. Ophthalmic Res. 47, 195–201 10.1159/000331992 22156553

[10] Deo M., Yu J.-Y., Chung K.-H., Tippens M. and Turner D.L. (2006) Detection of mammalian microRNA expression by in situ hybridization with RNA oligonucleotides. Dev. Dyn. 235, 2538–2548 10.1002/dvdy.20847 16736490

[11] Zhang L., Wang X. and Chen P. (2013) MiR-204 down regulates SIRT1 and reverts SIRT1-induced epithelial-mesenchymal transition, anoikis resistance and invasion in gastric cancer cells. BMC Cancer 13, 290 10.1186/1471-2407-13-290 23768087 PMC3710153

[12] Kassan M. et al. (2017) MicroRNA-204 promotes vascular endoplasmic reticulum stress and endothelial dysfunction by targeting Sirtuin1. Sci. Rep. 7, 9308 10.1038/s41598-017-06721-y 28839162 PMC5571183

[13] Guo L., Bai S.-P., Zhao L. and Wang X.-H. (2012) Astragalus polysaccharide injection integrated with vinorelbine and cisplatin for patients with advanced non-small cell lung cancer: effects on quality of life and survival. Med. Oncol. 29, 1656–1662 10.1007/s12032-011-0068-9 21928106

[14] Agyemang K. et al. (2013) Recent Advances in Astragalus membranaceus Anti-Diabetic Research: Pharmacological Effects of Its Phytochemical Constituents. Evid. Based. Complement. Alternat. Med. 654643, 1–9 10.1155/2013/654643 24348714 PMC3855992

[15] Wang N. et al. (2009) Astragalus polysaccharides decreased the expression of PTP1B through relieving ER stress induced activation of ATF6 in a rat model of type 2 diabetes. Mol. Cell. Endocrinol. 307, 89–98 10.1016/j.mce.2009.03.001 19524131

[16] Heller J.P., Kwok J.C.F., Vecino E., Martin K.R. and Fawcett J.W. (2015) A Method for the Isolation and Culture of Adult Rat Retinal Pigment Epithelial (RPE) Cells to Study Retinal Diseases. Front. Cell. Neurosci. 9, 449 10.3389/fncel.2015.00449 26635529 PMC4654064

[17] Oslowski C.M. and Urano F. (2011) Measuring ER Stress and the Unfolded Protein Response Using Mammalian Tissue Culture System. Methods Enzymol. 490, 71–92 10.1016/B978-0-12-385114-7.00004-0 21266244 PMC3701721

[18] Hetz C., Chevet E. and Harding H.P. (2013) Targeting the unfolded protein response in disease. Nat. Rev. Drug Discov. 12, 703–719 10.1038/nrd3976 23989796

[19] Shimazawa M. et al. (2007) Involvement of ER stress in retinal cell death. Mol. Vis. 13, 578–587 17438523 PMC2652022

[20] Cheung N., Mitchell P. and Wong T.Y. (2010) Diabetic retinopathy. Lancet 376, 124–136 10.1016/S0140-6736(09)62124-3 20580421

[21] Yau J.W.Y et al. (2012) Global Prevalence and Major Risk Factors of Diabetic Retinopathy. Diabetes Care 35, 556–564 10.2337/dc11-1909 22301125 PMC3322721

[22] Simó R., Villarroel M., Corraliza L., Hernández C. and Garcia-Ramírez M. (2010) The Retinal Pigment Epithelium: Something More than a Constituent of the Blood-Retinal Barrier–Implications for the Pathogenesis of Diabetic Retinopathy. J. Biomed. Biotechnol. 2010, 1–15 10.1155/2010/190724 PMC2825554 20182540

[23] Wei Z., Weng S., Wang L. and Mao Z. (2018) Mechanism of Astragalus polysaccharides in attenuating insulin resistance in Rats with type 2 diabetes mellitus via the regulation of liver microRNA-203a-3p. Mol. Med. Rep. 17, 1617–1624 29257218 10.3892/mmr.2017.8084 PMC5780102

[24] Gu C. et al. (2015) Astragalus polysaccharides affect insulin resistance by regulating the hepatic SIRT1-PGC-1α/PPARα-FGF21 signaling pathway in male Sprague Dawley rats undergoing catch-up growth. Mol. Med. Rep. 12, 6451–6460 10.3892/mmr.2015.4245 26323321 PMC4626146

[25] Huang Y.-F et al. (2016) Effects of Astragalus Polysaccharides on Dysfunction of Mitochondrial Dynamics Induced by Oxidative Stress. Oxid. Med. Cell. Longev. 9573291, 1-13 10.1155/2016/9573291 PMC4737051 26881048

[26] Hotamisligil G.S. (2010) Endoplasmic Reticulum Stress and the Inflammatory Basis of Metabolic Disease. Cell 140, 900–917 10.1016/j.cell.2010.02.034 20303879 PMC2887297

[27] Jing G., Wang J.J. and Zhang S.X. (2012) ER Stress and Apoptosis: A New Mechanism for Retinal Cell Death. Exp. Diabetes Res. 2012, 1–11 10.1155/2012/589589 PMC3246718 22216020

[28] Ma J.H., Wang J.J. and Zhang S.X. (2014) The Unfolded Protein Response and Diabetic Retinopathy. J. Diabetes Res. 2014, 1–14 10.1155/2014/160140 PMC4229964 25530974

[29] Zou F., Mao X.Q., Wang N., Liu J. and Ou-Yang J.P. (2009) Astragalus polysaccharides alleviates glucose toxicity and restores glucose homeostasis in diabetic states via activation of AMPK. Acta Pharmacol. Sin. 30, 1607–1615 10.1038/aps.2009.168 19960007 PMC4007496

[30] Ke B. et al. (2017) Astragalus polysaccharides attenuates TNF-α-induced insulin resistance via suppression of miR-721 and activation of PPAR-γ and PI3K/akt in 3T3-L1 adipocytes. Am. J. Transl. Res. 9, 2195–2206 28559971 PMC5446503

[31] Sun Q. et al. (2019) Protective Effects Of Astragalus Polysaccharides On Oxidative Stress In High Glucose-Induced Or SOD2-Silenced H9C2 Cells Based On PCR Array Analysis. Diabetes. Metab. Syndr. Obes. 12, 2209–2220 10.2147/DMSO.S228351 31695464 PMC6821059

[32] Sun S. et al. (2017) The effect of Astragalus polysaccharides on attenuation of diabetic cardiomyopathy through inhibiting the extrinsic and intrinsic apoptotic pathways in high glucose -stimulated H9C2 cells. BMC Complement. Altern. Med. 17, 310 10.1186/s12906-017-1828-7 28610566 PMC5470251

[33] Ooi C.Y. et al. (2018) Network modeling of microRNA-mRNA interactions in neuroblastoma tumorigenesis identifies miR-204 as a direct inhibitor of MYCN. Cancer Res. 78, 3122–3134 29610116 10.1158/0008-5472.CAN-17-3034

[34] Cai K.T. et al. (2019) Expression and potential molecular mechanisms of miR-204-5p in breast cancer, based on bioinformatics and a meta-analysis of 2,306 cases. Mol. Med. Rep. 19, 1168–1184 30569120 10.3892/mmr.2018.9764 PMC6323248

[35] López-González M.J., Soula A., Landry M. and Favereaux A. (2018) Oxaliplatin treatment impairs extension of sensory neuron neurites in vitro through miR-204 overexpression. Neurotoxicology 68, 91–100 10.1016/j.neuro.2018.07.009 30031110

[36] Yang F. et al. (2018) An androgen receptor negatively induced long non-coding RNA ARNILA binding to miR-204 promotes the invasion and metastasis of triple-negative breast cancer. Cell Death Differ. 25, 2209–2220 10.1038/s41418-018-0123-6 29844570 PMC6261952

[37] Li R., Zhu H., Yang D., Xia J. and Zheng Z. (2019) Long noncoding RNA lncBRM promotes proliferation and invasion of colorectal cancer by sponging miR-204-3p and upregulating TPT1. Biochem. Biophys. Res. Commun. 508, 1259–1263 10.1016/j.bbrc.2018.12.053 30563768

[38] Fan C. et al. (2019) Circular RNA circ KMT2E is up-regulated in diabetic cataract lenses and is associated with miR-204-5p sponge function. Gene 710, 170–177 10.1016/j.gene.2019.05.054 31153886

[39] Wu S. et al. (2017) Effect of dietary Astragalus Polysaccharide supplements on testicular miRNA expression profiles and enzymatic changes of breeder cocks. Sci. Rep. 7, 38864 10.1038/srep38864 28054553 PMC5214674

