# Expression of Concern: Astragalus polysaccharide attenuates metabolic memory-triggered ER stress and apoptosis via regulation of miR-204/SIRT1 axis in retinal pigment epithelial cells

**DOI:** 10.1042/BSR20192121_EOC

**Published:** 2026-08-12

**Authors:** Qing-Hua Peng, Ping Tong, Li-Min Gu, Wen-Jie Li

**Affiliations:** 1 Department of Ophthalmology and Otorhinolaryngology in Chinese Medicine, Hunan University of Chinese Medicine and Hunan Key Laboratory, Changsha 410208, P.R. China; 2 Department of Ophthalmology, The Second Xiangya Hospital, Central South University, Changsha 410011, P.R. China; 3 Department of Ophthalmology, Third Affiliated Hospital of Second Military Medical University, Shanghai 200438, P.R. China; 4 College of Integrated Traditional Chinese and Western medicine of Hunan University of Chinese Medicine, Changsha 410013, P.R. China

**Keywords:** Apoptosis, Astragalus polysaccharides, ER stress, Metabolic memory, miR-204, SIRT1

The Editorial Office has been made aware of potential issues surrounding the scientific validity of this paper and is hence issuing a permanent expression of concern to notify readers.

It has been noted that the paper reports use of inappropriate primers for the amplification of U6 snRNA. Additionally, a number of unexpected similarities have been identified within the images presented in this manuscript. Similarities noted between images are described below:

- Figure 2A ARPE-19 p-PERK bands appear similar to the PRPE p-PERK bands
- Figure 2A ARPE-19 PERK bands appear similar to the ARPE-19 IRE-1 bands following a horizontal flip
- Figure 2A PRPE p-IRE-1 bands appear similar to the PRPE Cleaved-ATF-6 bands
- Figure 3A OSM plot appears similar to Figure 7A HG→NG+ APS + shSIRT1 plot
- Figure 3A HG→NG+ 25 μg/mL APS plot appears similar to Figure 7A HG→NG+ APS + miR-204 NC plot
- Figure 3A HG→NG+ 50 μg/mL APS plot appears similar to Figure 7A HG plot
- Figure 4 ARPE-19 Cleaved-caspase-12 bands appear similar to ARPE-19 Cleaved-caspase-9 bands
- Figure 4 ARPE-19 Cleaved-caspase-12 bands appear similar to PRPE Cleaved-caspase-12 bands
- Figure 4 ARPE-19 Uncleaved PARP bands appear similar to the PRPE Uncleaved PARP bands
- Figure 4 PRPE Bcl-2 bands appear similar to the Cleaved-caspase-12 bands

The authors have been contacted for comment and have not provided a response or the requested raw data.

As the issues are unable to be resolved, this expression will remain published permanently or until further evidence arises.

