# Supplementary material for: Astragalus polysaccharide attenuates metabolic memory-triggered ER stress and apoptosis via regulation of miR-204/SIRT1 axis in retinal pigment epithelial cells
Source: Biosci Rep. 2020 Jan 21;40(1):BSR20192121. doi: 10.1042/BSR20192121 (PMC6974424; doi:10.1042/BSR20192121)
Supplement: Supplementary Figure S1 [file BSR-2019-2121_supp.pdf]

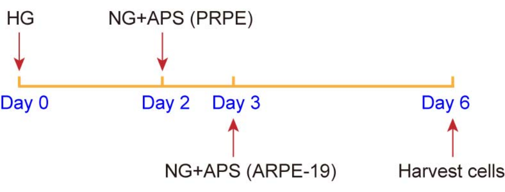
HG
NG+APS (PRPE)
Day 0
Day 2
Day 3
Day 6
NG+APS (ARPE-19)
Harvest cells

Supplementary figure 1. A schematic with the detailed treatment. For ARPE-19 cells, cells in temporary high glucose group were treated with high glucose (30 mM) for 3 days followed by normal glucose (5 mM) for 3 days. For PRPE cells, cells in temporary high glucose group were treated with high glucose for 2 days followed by normal glucose for 4 days. For APS treatment, APS (12.5, 25 and 50 μg/mL) was dissolved in normal glucose condition and added to cells whenever high glucose was replaced with normal glucose.
